# Supplementary material for: Sporadic Dissemination of tet(X3) and tet(X6) Mediated by Highly Diverse Plasmidomes among Livestock-Associated Acinetobacter
Source: Microbiol Spectr. 2021 Dec 1;9(3):e01141-21. doi: 10.1128/Spectrum.01141-21 (PMC8635130; doi:10.1128/Spectrum.01141-21)
Supplement: SUPPLEMENTAL FILE 1 — Supplemental material. Download SPECTRUM01141-21_Supp_1_seq12.pdf, PDF file, 6.5 MB [file spectrum01141-21_supp_1_seq12.pdf]

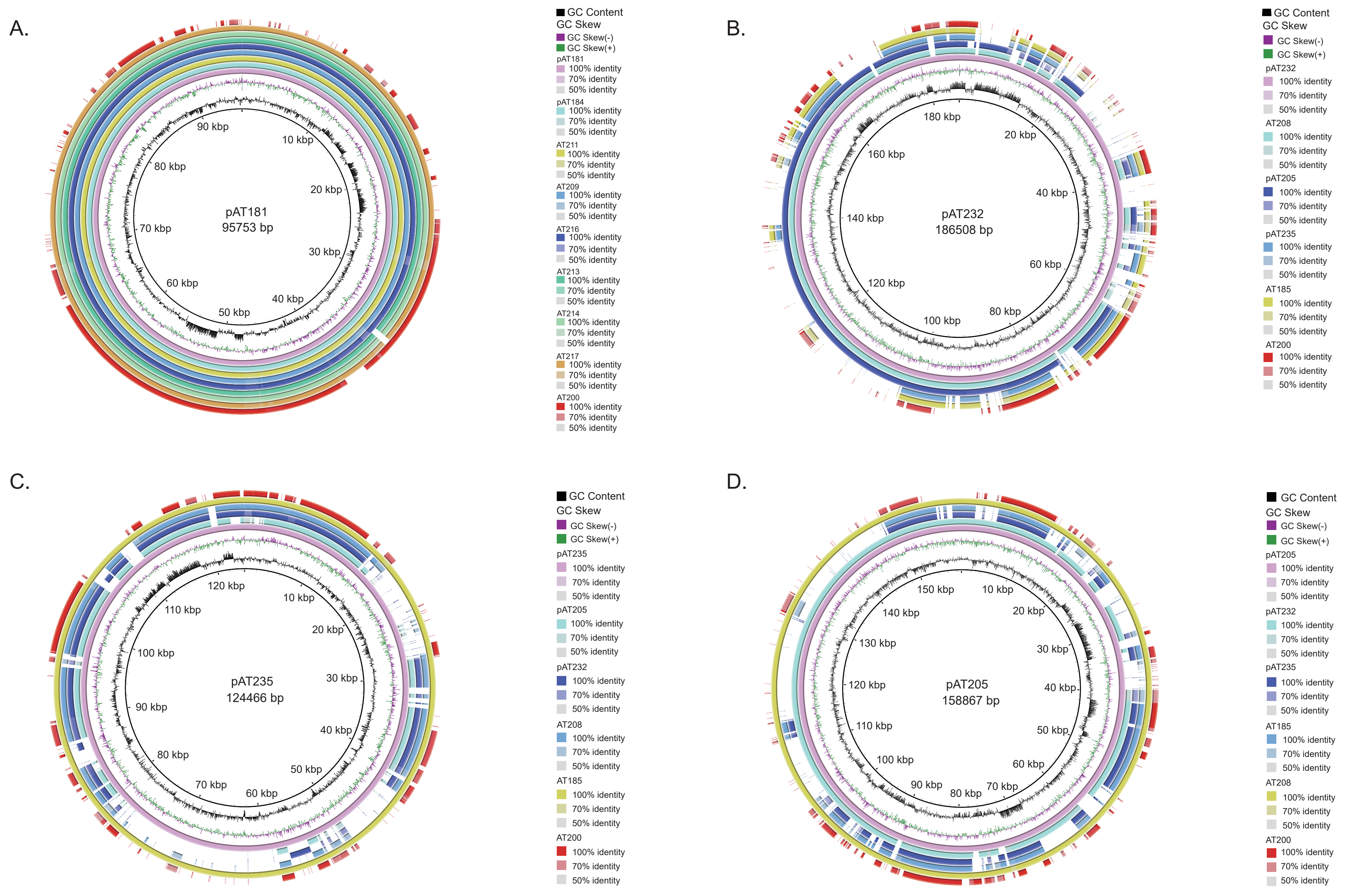

Supplementary figure 1. Comparative analysis of tet(X)-encoding plasmids in this study. (A) The inner ring represents the circularized tet(X3)-encoding plasmid pAT181 as reference; (B) The inner ring represents the circularized tet(X6)-encoding plasmid pAT232 as reference; (C) The inner ring represents the circularized tet(X6)-encoding plasmid pAT235 as reference; (D) The inner ring represents the circularized tet(X6)-encoding plasmid pAT205 as reference.

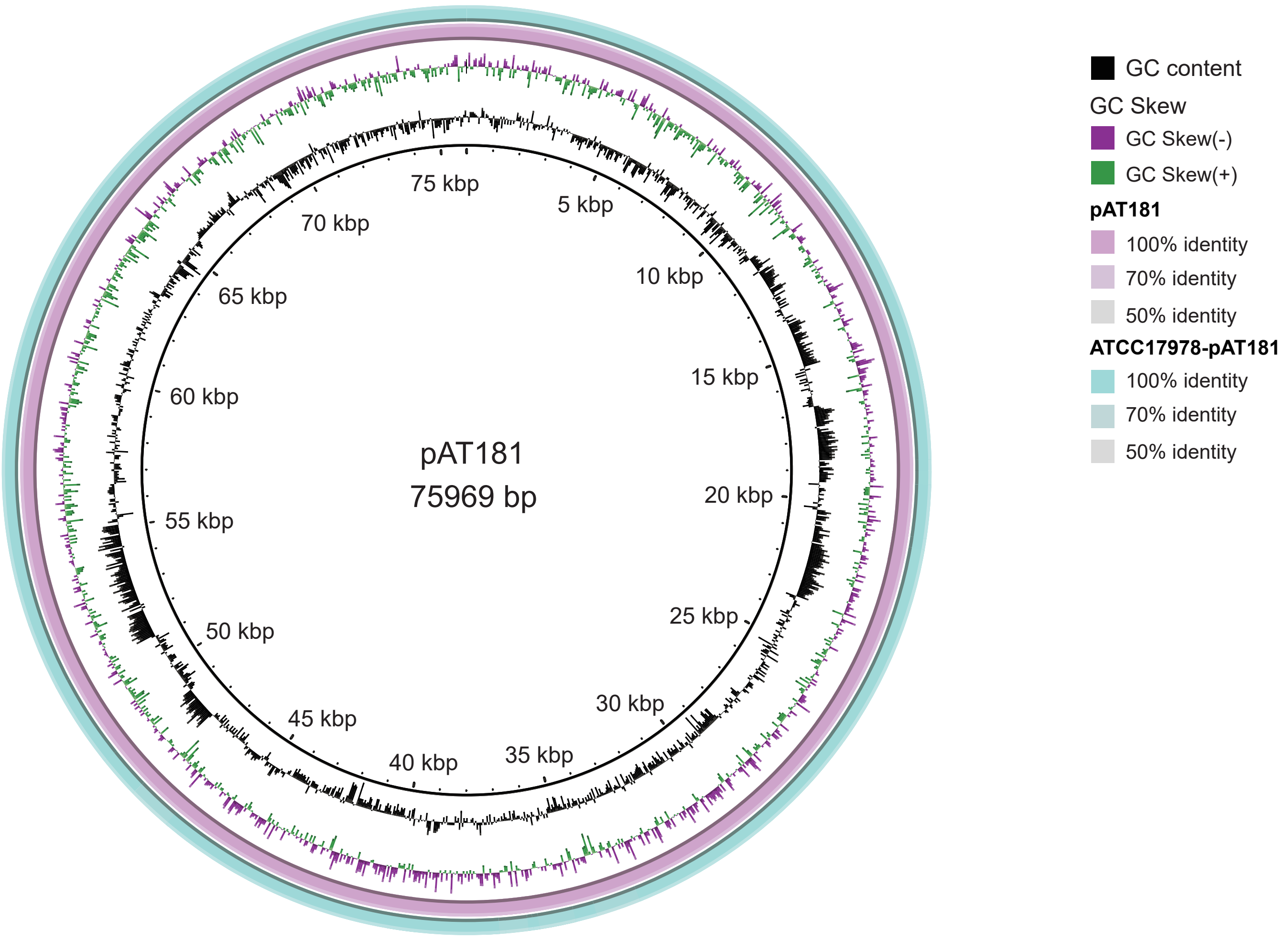

Supplementary figure 2. Verification of horizontal transfer of tet(X3)-encoding plasmid pAT181 in the transconjugant ATCC17978-pAT181. The inner ring represents pAT181 as reference. The outer ring represents the mapping result of exogenous DNA in the tansconjugant AB181 compared with the recipient strain ATCC17978.

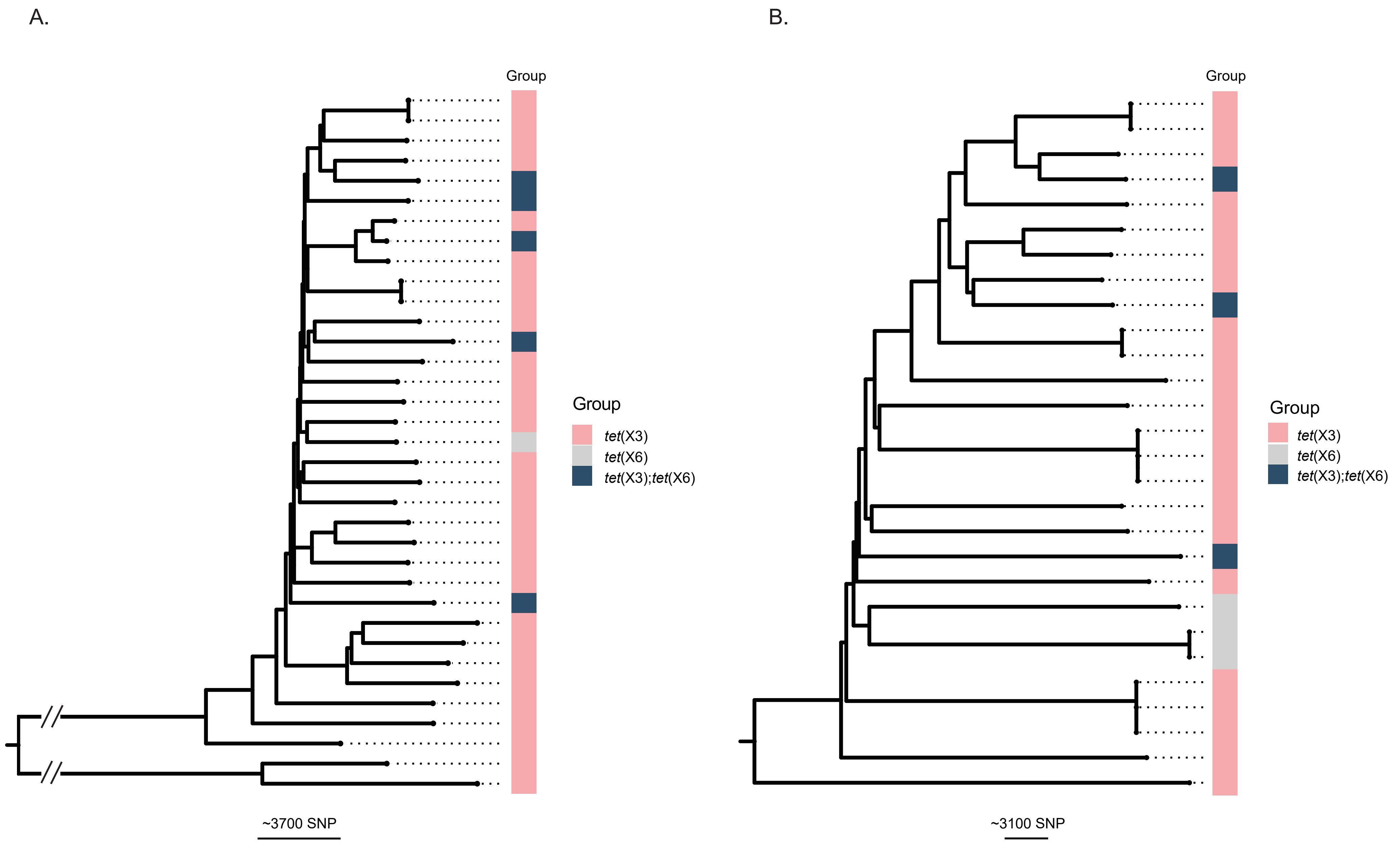

Supplementary figure 3. Phylogenetic analysis of tet(X3)/tet(X6)-encoding A. towneri (A) and A. variabilis (B) genomes retrieved from GenBank. The core-genome SNPs of tet(X)-encoding strains were used to generate the phylogenetic tree. The tree is mid-point rooted. The tet(X) genes (group), isolate source (host), sampling location (location) and years (date) of strains are exhibited at the right side of phylogenetic tree in different colors.

Table S1. Short-read and long-read sequenced isolates collected in this study.

Sample ID	Sequencing platform	Number of reads	No. of contigs >500bp	No. of contigs >= 1kb	Assembly length (bp)	N50 (bp)
ZJ180	Illumina	7055328	98	89	2817934	71674
ZJ182	Illumina	8431912	54	48	2763184	121815
ZJ185	Illumina	6701128	53	48	2710243	130902
ZJ200	Illumina	9751606	49	44	2800022	189532
ZJ202	Illumina	11228728	68	61	2723892	68975
ZJ208	Illumina	8286854	79	59	2752293	117964
ZJ209	Illumina	9401360	87	70	2839455	77345
ZJ211	Illumina	10598468	91	72	2841347	77345
ZJ213	Illumina	7430552	88	72	2835957	77346
ZJ214	Illumina	9348336	88	71	2835746	77346
ZJ215	Illumina	7646804	74	68	2938767	86704
ZJ216	Illumina	8940680	85	67	2839563	78054
ZJ217	Illumina	11986108	87	70	2836553	78336
ZJ286	Illumina	8246802	62	49	3895768	159846
ZJ291	Illumina	7984526	103	82	3917645	135379
ZJ295	Illumina	8564982	61	50	3894412	159960
ZJ181	llumina & nanopore	10463826 & 182398	5	5	2944896	2849143
ZJ183	llumina & nanopore	10971212 & 143527	4	4	2839791	2821396
ZJ184	llumina & nanopore	8823122 & 164141	5	5	2944891	2849142
ZJ199	llumina & nanopore	5051594 & 46377	1	1	3048990	3048990
ZJ205	llumina & nanopore	9801474 & 113173	5	5	2829067	2660496
ZJ232	llumina & nanopore	8397580 & 107498	2	2	2997551	2811043
ZJ235	llumina & nanopore	8407100 & 90443	2	2	2853583	2729117

Table S2. The ARGs carried by *E. stercoris* and *M. odoratimimus* isolates collected in this study

Strains		ARGs			
		**Macrolide**	**Tetracycline**	**Beta-lactam**	**Sulphonamide**
E. stercoris	**ZJ180**	*mef* (C), *mph* (G)	*tet* (X2)	$bla_{\text{EBR-1}}$	
	**ZJ182**		*tet* (X2), *tet* (X14)	$bla_{\text{EBR-1}}$	
	**ES183**		*tet* (X2), *tet* (X2), *tet* (X14)	$bla_{\text{EBR-1}}$	
	**ZJ202**		*tet* (X2)	$bla_{\text{EBR-1}}$	
	**ZJ215**	*mef* (C), *mph* (G)	*tet* (X2)	$bla_{\text{EBR-1}}$	
M. odoratimimus	**ZJ286**	*ereD* , *ereD*	*tet* (X2), *tet* (36)	$bla_{\text{MUS-1}}$	
	**ZJ291**	*ereD* , *ereD*	*tet* (X2), *tet* (36)	$bla_{\text{MUS-1}}$	*sul2*
	**ZJ295**	*ereD* , *ereD*	*tet* (X2), *tet* (36)	$bla_{\text{MUS-1}}$	

Table S3. Transposition of tet(X3)-carrying plasmid increased resistance to tetracyclines

Strains	MIC (mg/L)						
	TGC	TET	OTC	CTC	DMC	DOX	ERV
**ATCC17978 (recipient)**	0.06	1	2	0.25	0.25	0.25	0.06
**AB181 (transconjugant)**	8 (128x)	>128 (>128x)	>128 (>64x)	>128 (>512x)	>128 (>512x)	16 (64x)	16 (256x)

Abbreviation: TGC, Tigecycline; TET, Tetracycline; OTC, Oxytetracycline; CTC, Chlortetracycline; DMC, Demeclocycline; DOX, Doxycycline; ERV, Eravacycline.
